# Bilateral Branch Retinal Vein Occlusion after mRNA-SARS-CoV-2 Booster Dose Vaccination

**DOI:** 10.3390/jcm12041325

**Published:** 2023-02-07

**Authors:** Matteo Gironi, Rossella D’Aloisio, Tommaso Verdina, Benjamin Shkurko, Lisa Toto, Rodolfo Mastropasqua

**Affiliations:** 1 Ophthalmology Clinic, University of Modena and Reggio Emilia, Azienda Ospedaliero-Universitaria di Modena, 41125 Policlinico, Italy; 2 Ophthalmology Clinic, Department of Medicine and Science of Ageing, University G. D’Annunzio Chieti-Pescara, 66100 Chieti, Italy; 3 Ophthalmology Clinic, Department of Medicine and Surgery, University of Parma, 43121 Parma, Italy

**Keywords:** COVID, SARS-CoV-2, retinal vein occlusion, RVO, vaccination, branch retinal vein occlusion

## Abstract

Purpose: We report a case of a patient with a bilateral branch retinal vein occlusion (BRVO) 24 h after a booster vaccination with the mRNA-1237 vaccine. Observations: Fluorescein angiography, performed at three weeks follow-up, showed vascular leakage and blockage, corresponding to hemorrhage areas associated with ischemic areas in the macula and along the arcades involved in the occlusion. Conclusions: The patient was scheduled for urgent injections of intravitreal ranibizumab and laser photocoagulation of the ischemic areas. To the best of our knowledge, this is the first case described of concomitant bilateral RVO after COVID-19 vaccination. The rapid onset of the side effects in a patient with multiple risk factors for thrombotic events suggests that vulnerable microvascular conditions require detailed investigations before administration of a COVID-19 vaccine.

## 1. Introduction

Vaccines are essential to limit the social impact of the COVID-19 pandemic. By January 2022, more than 9.5 billion doses of COVID-19 vaccines were administered worldwide.

Vaccine-related serious side effects reported in the literature have varied and have included cerebral venous sinus thrombosis and immune thrombotic thrombocytopenia [1]. Several types of ocular manifestations have also been reported [2].

To the best of our knowledge, no report on simultaneous bilateral retinal vein occlusion (RVO) consequent to vaccination has been described until now. Here, we report a case of a bilateral branch retinal vein occlusion (BRVO) after a booster vaccination with the mRNA-1237 vaccine (Moderna).

## 2. Case Report

A 50-year-old Caucasian man was referred to our Ophthalmological Emergency Service for painless sudden vision loss in both eyes, onset 24 h after a booster dose with the mRNA-1237 vaccine. He had received the mRNA-vaccine BNT162b2 for the first two vaccination doses, without side effects. No previous infection of SARS-CoV-2 was reported. Past medical history included an emergency hospitalization in 2020 for mild acute heart failure (NYHA II) in newly diagnosed dilated cardiomyopathy and arterial hypertension. At discharge, a multi-pharmacological antihypertensive treatment was set. A family history of heart attack was reported (father died at 50 of myocardial infarction). The patient is a non-smoker with mild obesity (BMI = 33.4).

During the ophthalmological evaluation, the patient was in treatment with Valproate and Lurasidone for psychotic syndrome. The latest previous blood tests did not show pathologically altered values, while the ECG revealed a modest alteration due to ventricular overload.

**Figure 1:**
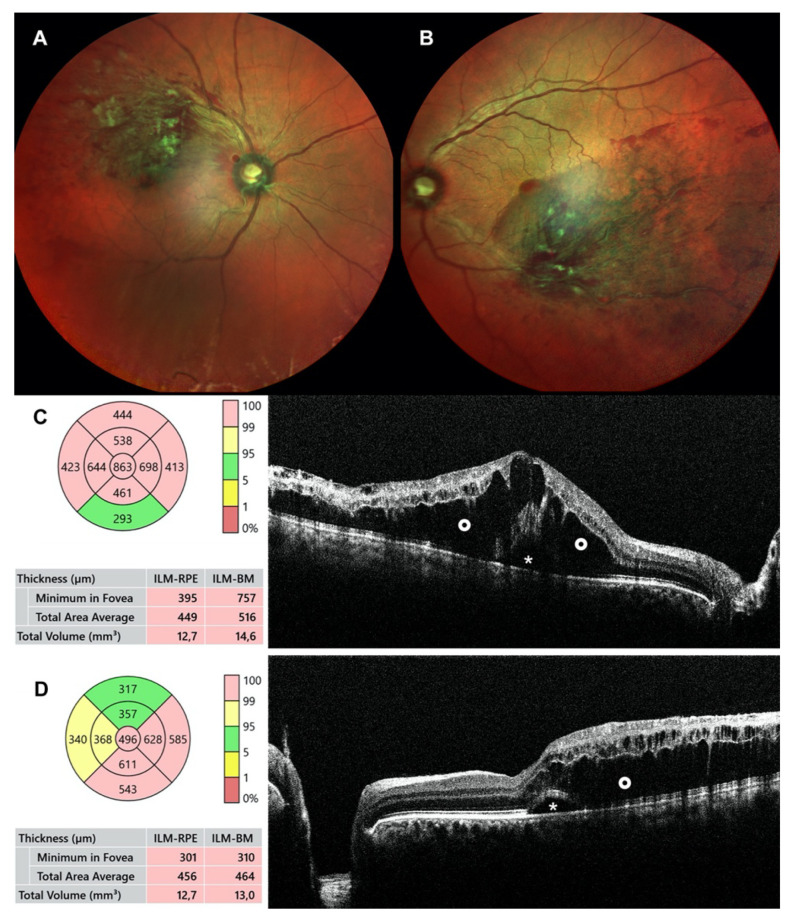
Baseline posterior pole multicolor image and optical coherence tomography of both eyes: (**top**) Fundus photographs showing widespread hemorrhages and axonal congestion upstream of the venous occlusion of superotemporal branch vein in the right eye (**A**) and inferotemporal branch vein in the left eye (**B**); (bottom) OCT macula (Macula 3D mode) shows a significant cystoid macular edema and intraretinal fluid (O), associated with subfoveal neuroretinal detachment (*) in both eyes, with a central macular thickness of 863 μm for the right eye (**C**) and 496 μm for the left eye (**D**).

The ophthalmic evaluation revealed a best-corrected visual acuity of 20/200 in the right eye and 20/28 in the left eye. Intraocular pressure and anterior segment exams were normal in both eyes. A fundus examination showed congested tortuous veins associated with flame hemorrhages and cotton wool spots of superior-temporal arcade in the right eye and inferior-temporal arcade in the left eye. Pathological signs were more extensive in the right eye than in the left eye. (Figure 1A,B). High-resolution optical coherence tomography, performed at presentation, revealed significant macular edema in both eyes, as shows Figure 1.

The last previous eye examination was 6 months before vaccination, and it reported a retinal vascular tree within the age range, with no other relevant alterations.

Extensive screening blood examinations, including full blood cell counts and differential with peripheral blood smear, platelet count, electrolytes, lipid profile, fasting glycemia, iron tests, liver enzymes, serum protein, lipid profile, serum bilirubin, and serum creatinine, revealed only a mild alteration of liver functionalities. C-reactive protein was 0.3 mg/L (range 0–0.7) and erythrocyte sedimentation rate was 3 mm/h (range 2–28). Other screening blood examinations were performed, including thyroid hormones, vitamin B12, folate, serum homocysteine, glycated hemoglobin (HbA1c), anti-cardiolipin antibodies, thrombophilia screen, treponema pallidum screening, cytomegalovirus IgM-IgG, and serum HIV, which were all unremarkable.

**Figure 2:**
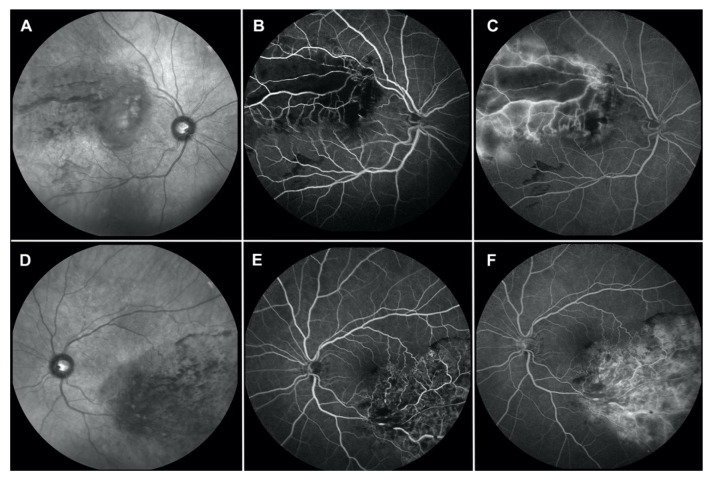
Fundus autofluorescence (FAF) and fluorescein angiography (FA) of both eyes at three weeks follow-up. FAF of the right (**A**) and left (**D**) eyes hyper-autofluorescence corresponding to intraretinal fluid near the fovea and hypo-autofluorescence as a result of blockage intraretinal hemorrhage. FA shows ischemic branch retinal vein occlusion with blockage corresponding to the retinal hemorrhage in early phases ((**B**), right and (**E**), left). Late phases showed extensive leakage in the area of branch retinal vein occlusion and clinically significant macular edema ((**C**), right and (**F**), left). Arm-retina time, 16 s; Right eye arteriovenous transit time, 42 s; Left eye arteriovenous transit time, 32 s.

Fluorescein angiography, performed at three weeks follow-up, showed vascular leakage and blockage, corresponding to the hemorrhage areas associated with ischemic areas in the macula and along the arcades involved in the occlusion (Figure 2).

The patient was scheduled for urgent injections of intravitreal ranibizumab and laser photocoagulation of ischemic areas.

## 3. Discussion

BRVO is the second most common retinal vascular disease. Despite this, the occurrence of concurrent bilateral BRVO is uncommon.

Here, we reported a case of bilateral BRVO likely due to booster dose vaccination. To the best of our knowledge, this is the first case described of concomitant bilateral RVO after COVID-19 vaccination.

Undoubtedly, our patient presented underlying conditions that moderately exacerbated thrombogenicity, such as hypertension and obesity, even if his blood cell counts and hemostasis tests were unremarkable [3].

In the literature, a few case reports of unilateral RVO after COVID-19 vaccination have been reported [2,3,4,5]. Nonetheless, one of these depicted a concomitant central retinal artery and vein occlusion after the second dose [6].

Recently, two case series on ocular complications after SARS-CoV-2 vaccination have shown that more than 50% of eyes who developed RVO had received an mRNA vaccine. The median time between vaccination and symptom exacerbation was 2 days [2,4]. No bilateral cases were identified in these series.

A case report of bilateral branch retinal vein occlusions secondary to sodium valproate therapy has been reported in the literature [7]. Sodium valproate can induce increased levels of serum homocysteine (HC), which is an independent factor for vascular events [8]. Despite this, the HC levels detected in the patient were within the range of normality (9 μmol/L).

Although the pathogenesis mechanism requires further study, possible hypotheses on such an adverse event are focusing on spike proteins as triggering an atypical procoagulant and proinflammatory response, especially in eyes more vulnerable to microvascular dysfunction [4].

## 4. Conclusions

In conclusion, here, we report a case where a drug-related vascular event relationship is very strong, because of concomitant manifestation in both eyes. The rapid onset of the side effects in a patient with multiple risk factors for thrombotic events suggests that vulnerable microvascular conditions require detailed investigations before administration of a COVID-19 vaccine.

## Data Availability

All data are available on a reasonable request to the corresponding author.
